# Divergent RNA Localisation Patterns of Maternal Genes Regulating Embryonic Patterning in the Butterfly *Pararge aegeria*

**DOI:** 10.1371/journal.pone.0144471

**Published:** 2015-12-03

**Authors:** Jean-Michel Carter, Melanie Gibbs, Casper J. Breuker

**Affiliations:** 1 Evolutionary Developmental Biology Research Group, Department of Biological and Medical Sciences, Faculty of Health and Life Sciences, Oxford Brookes University, Gipsy Lane, Headington, Oxford, OX3 0BP, United Kingdom; 2 NERC Centre for Ecology & Hydrology, Maclean Building, Benson Lane, Crowmarsh Gifford, Wallingford, Oxfordshire, OX10 8BB, United Kingdom; University of Otago, NEW ZEALAND

## Abstract

The maternal effect genes responsible for patterning the embryo along the antero-posterior (AP) axis are broadly conserved in insects. The precise function of these maternal effect genes is the result of the localisation of their mRNA in the oocyte. The main developmental mechanisms involved have been elucidated in *Drosophila melanogaster*, but recent studies have shown that other insect orders often diverge in RNA localisation patterns. A recent study has shown that in the butterfly *Pararge aegeria* the distinction between blastodermal embryonic (i.e. germ band) and extra-embryonic tissue (i.e. serosa) is already specified in the oocyte during oogenesis in the ovariole, long before blastoderm cellularisation. To examine the extent by which a female butterfly specifies and patterns the AP axis within the region fated to be the germ band, and whether she specifies a germ plasm, we performed *in situ* hybridisation experiments on oocytes in *P*. *aegeria* ovarioles and on early embryos. RNA localisation of the following key maternal effect genes were investigated: *caudal* (*cad*), *orthodenticle* (*otd*), *hunchback* (*hb*) and four *nanos* (*nos*) paralogs, as well as *TDRD7* a gene containing a key functional domain (OST-HTH/LOTUS) shared with *oskar*. TDRD7 was mainly confined to the follicle cells, whilst *hb* was exclusively zygotically transcribed. RNA of some of the *nos* paralogs, *otd* and *cad* revealed complex localisation patterns within the cortical region prefiguring the germ band (i.e. germ cortex). Rather interestingly, *otd* was localised within and outside the anterior of the germ cortex. Transcripts of *nos-O* formed a distinct granular ring in the middle of the germ cortex possibly prefiguring the region where germline stem cells form. These butterfly RNA localisation patterns are highly divergent with respect to other insects, highlighting the diverse ways in which different insect orders maternally regulate early embryogenesis of their offspring.

## Introduction

In the main, the insect body plan consists along the anterior-posterior (AP) axis of a head, thorax and abdomen [1–3]. *Drosophila melanogaster* studies on the developmental mechanisms underlying such patterning during embryogenesis, and crucially the role of maternal effect genes therein, have become iconic (e.g. [4]). Furthermore, primordial germ line stem cells (PGCs) of the *Drosophila* embryo are already maternally specified in the form of a germ plasm, which is to some extent integrated with AP patterning [5]. In recent years studies on other insects have revealed that although maternal regulation *per se* of early embryogenesis is shared, the details often differ [3], and the presence of a germ plasm is by no means widespread [5].

**Fig 1:**
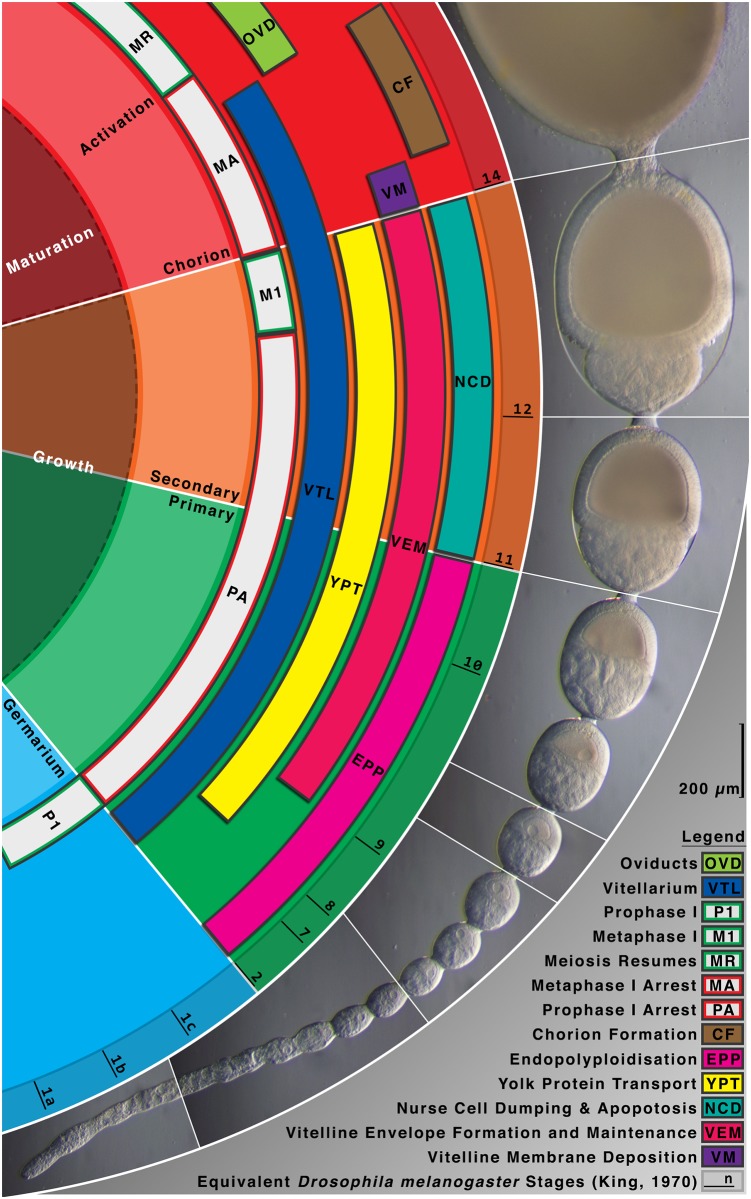
Oogenesis progression in *Pararge aegeria*. *Pararge aegeria* ovaries consist of 8 ovarioles [6]. The diagram illustrates the morphology of a single *P*. *aegeria ovariole* annotated with the approximate equivalent *Drosophila melanogaster* oogenesis stages (i.e. 1 to 14) [56] on the basis of relative size, morphological characteristics and position of the nucleus (abbreviations explained in figure). Cellular processes (e.g. meiosis I and II) and ovarian regions (e.g. oviducts) relevant to oogenesis are also annotated. Progression of oocytes beyond the vitellarium is scaled to fit. The nurse cells have fully degenerated and the oocytes in this region will enter the oviducts and be ready for fertilisation and laying, upon which embryogenesis commences.

Broadly speaking, differences in maternal effect gene regulation arise as a result of the type of oogenesis (e.g. panoistic versus meroistic ovaries), the amount of yolk included (i.e. maternal reproductive strategies), and the germ-band type of the embryo (short, intermediate or long germ) [3]. Butterflies are in many respects like *Drosophila* in that they produce yolk containing eggs in polytrophic meroistic ovaries [6]. Unique to meroistic ovarioles is the presence of nurse cells (sister cells derived from the same germ cell) connecting at the anterior of each oocyte (polytrophic) or the ovariole (telotrophic) [7]. Each polytrophic follicle (consisting of nurse cells and oocyte) is enclosed by follicle cells. As the follicle progresses through the vittelarium and the oocyte matures, the nurse and follicle cells eventually die off through apoptosis. The fully mature oocyte passes through the oviduct where it is fertilised and then laid on a suitable host plant (Fig 1). The nurse cells are transcriptionally very active producing crucial proteins and mRNA of maternal effect genes and the polytrophic arrangement enables these to be transferred directly into the transcriptionally inactive oocytes within each follicle.

**Fig 2:**
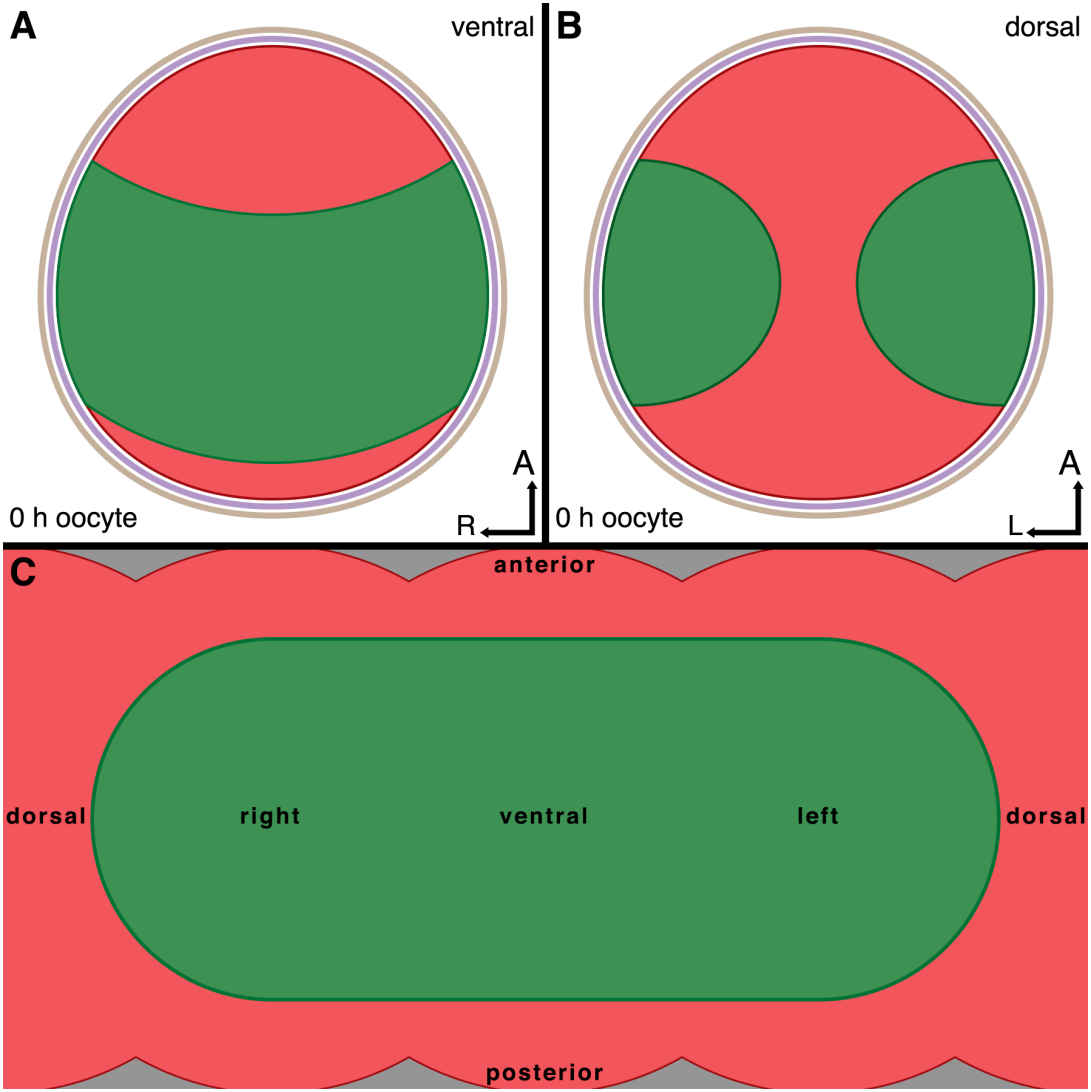
Overview of embryonic and extraembryonic fated regions in the cortex of *Pararge aegeria* oocytes, Schematic ventral **(A)** and dorsal **(B)** views of the cortical regions fated to become the embryonic germ band **(green)** or extraembryonic tissue **(red)** in a mature oocyte surrounded by a vitelline membrane **(purple)** and chorion **(brown)**. Simplified 2D map of the oocyte cortex and cortical domains **(C)**. All times are in hours after egg-laying.

Ditrysian embryos, show characteristics of both intermediate and long germ insects [8,9]. Both *Bombyx mori* and butterflies belong to the Ditrysia, a derived clade within Lepidoptera [1]. Despite some studies on *B*. *mori* embryology [9–11], maternal RNA localisation in Ditrysia in general, and butterflies in particular, has received little attention. Ovarian and maternal effect gene transcriptomes from Speckled Wood butterflies *Pararge aegeria* [6,12] have suggested that butterflies show significant divergence in the maternal effect genes used, compared to *Drosophila* in particular and other insects in general. Moreover, during butterfly oogenesis females localise *ShxC* transcripts in the oocyte exactly where the extra-embryonic tissue (i.e. serosa) will form, resulting in one of the most complex intracellular RNA localisation patterns ever documented [1]. The remaining area reserved for the germ band is a wide semi-circular band (see Fig 2 and [1]). Early embryos in Ditrysia are characterised by a wide germ band, which will first contract (horizontally) and then elongate (vertically) in later stages [13,14] (also see S8 Fig in [1]).

Nothing is known about where female butterflies localise RNA transcripts in the region fated to be the germ band, specifically those patterning the embryo along the AP axis, nor whether butterflies have a maternally specified germ plasm [1,5,6,15,16].

In *Drosophila*, *cad* transcripts are distributed quite evenly throughout the oocyte, but *cad* translation is restricted to the posterior of the syncytial embryo as a result of an AP gradient of Bcd protein [4]. Regulation of *cad* translation in order to achieve a posterior gradient and localisation of Cad protein may not be necessary if *cad* transcripts have already been precisely maternally localised posteriorly. This has been observed in a number of long germ hymenopteran species, such as *Nasonia vitripennis* and *Apis mellifera* [17,18].

As *bcd* is unique to *Drosophila* [19], it has been proposed that *otd* is the ancestral anterior determinant in insects, not least because it shares a K50 Homeodomain with *bcd* ([20]; but see [21,22]). It has also been hypothesized that KH domain factors such as *mex-3* may play an ancestral anterior patterning role through *cad* repression based on work in *Tribolium castaneum* [23].

Both maternal *otd* and *cad* transcripts are localised in the *B*. *mori* oocyte and may guide embryonic AP patterning [11], but rather curiously the Cad protein gradient established during gastrulation does not appear to be initially established maternally [24]. Transcripts of both *otd* and *cad* have been detected in *P*. *aegeria* oocytes [6], but their localisation patterns remain uncharacterised.

In *Drosophila*, *hunchback* transcripts are involved in anterior patterning, both maternally and zygotically [3]. In other insects, maternal *hb* is important for embryonic AP patterning, whether maternally provided as mRNA or protein [11,25]. Lepidoptera appear to be an exception [25], and indeed no maternal *hb* transcripts have been detected in butterfly ovaries and oocytes [6]. In this study we will investigate whether butterflies have indeed dispensed with a maternal contribution to the Hb gradient in the embryo.

*Nanos* holds both a role in germ plasm differentiation and posterior patterning in *D*. *melanogaster* [26]. In *B*. *mori* PGCs appear to develop in a mid-ventral position in the germ disk after blastoderm formation [10]. This spatio-temporal segregation constrains possibilities for any overlap in genes regulating germ plasm differentiation and posterior patterning [10,14]. Four, functionally different, *nos* paralogs have been identified in *B*. *mori* and *P*. *aegeria* (*nos-M*, *-O*, *-P* and–*like (also called—N)*) [6,10]. Although the existence of a germ plasm in Ditrysia is uncertain, *B*. *mori nos-O* RNA granules have been found distributed along the ventral midline, partially overlapping with where the PGCs will form [10]. These *nos* paralogs, with the exception of *nos-P*, are expressed during oogenesis in both *B*. *mori* and *P*. *aegeria* [6,10].

*Pararge aegeria* does not possess an *osk* ortholog [6], a gene suggested to have been co-opted for germ plasm formation in holometabolous insects [5,15]. However a number of genes involved in polar granule formation in insects with a germ plasm have been shown to be expressed in *P*. *aegeria* during oogenesis [6]. Interestingly, among these is TUDOR-domain containing gene *TDRD7*, which shares the OST-HTH/LOTUS functional domain with *osk* [5,27–29]. We will therefore also investigate whether maternal RNA localisation of the *nos* paralogs and *TDRD7* correspond to where the PGCs will develop, and thus form a basis for maternal germ plasm-like specification in butterflies.

In the present study we examine the spatio-temporal expression patterns of key maternal effect genes [3,5,6] in oocytes in *P*. *aegeria* ovarioles and in early embryos using whole mount *in situ* hybridisation (WMISH). Compared to other studied insects, previous work on *P*. *aegeria* has indicated divergence with respect to maternal effect gene expression [6], and the unusual maternal specification of a semi-circular area in the oocyte fated to be the germ band [1]. It is therefore to be expected that butterflies, or Ditrysia in general, have evolved novel ways to maternally regulate patterning along the AP axis in the region fated to be the germ band. We therefore aimed to determine the extent of cortical localisation of maternal effect genes likely to be involved in AP patterning (*cad*, *otd*, *hb* and *nos* paralogs). Finally, butterflies have been argued to not specify a germ plasm since they do not have the gene *osk*, although there is the possibility that they may have evolved a novel way to do so. In order to determine whether there is any evidence for germ plasm-like specification in butterflies we investigated the localisation of *TDRD7* and *nos* paralogs.

## Materials and Methods

### Butterfly rearing and sample collection

Ovarioles (from 4-day old females), eggs and embryos (10h and 12h old) were collected from outbred laboratory stock butterflies (cf. [1,6] for details). Furthermore, in order to illustrate where *cad* was expressed once segmentation had been established, 25 h old embryos (corresponding roughly to the arthropod phylotypic stage) were stained for the segment polarity gene *engrailed* (*en*), as the segmentation marker, as well as *cad*. RNA from eggs and ovaries was obtained using a TRI-Reagent extraction followed by RNeasy purification as detailed previously [6]. cDNA was generated using the Verso RT Kit (Thermo Fisher, Surrey, UK) and stored at −20°C. Ovary and egg cDNA was pooled for subsequent riboprobe preparation.

### Riboprobe generation

Regions targeted (S1 Fig) were amplified by PCR and modified in a second PCR using primers with a T7 overhang. All primers used for riboprobe generation are presented in S1 Table. All riboprobes were synthesised from final PCR products flanked with a T7 promoter (3’ for antisense) using a T7 DIG RNA labelling Kit (Roche Applied Science, Penzberg, Germany) as per the manufacturer’s instructions. The resulting riboprobes were purified with an RNeasy Kit (Qiagen, Hilden, Germany) and stored at -20°C.

### Whole mount *in situ* hybridisation

The whole mount *in situ* hybridisation (WMISH) protocol used is described in detail elsewhere [1]. In summary, eggs were gently dechorionated and thoroughly washed before fixation, while ovaries were directly fixed. The fixation was allowed to proceed overnight at 4°C before dehydration. Samples were stored at -20°C for a few days. Before hybridisation, samples were rehydrated and digested with proteinase-K. The samples were fixed a second time, washed and incubated in pre-hybridisation solution. The Hybridisation solution (50% Deionised formamide, 5x SSC, 0.02% Tween 20, 100 μg/ml denatured Yeast tRNA, 2 mg/ml Glycine) containing 100 ng/μl of riboprobe was swiftly applied to the samples minimizing temperature fluctuations. Hybridisation was allowed to proceed overnight at 55°C. The hybridised samples were washed and blocked (Roche Applied Science, Penzberg, Germany) for 30 min before anti-DIG antibody incubation at room temperature for 3–4 h. Excess antibody was washed thoroughly including a final overnight wash at 4°C. Staining was developed in Alkaline Phosphatase buffer with NBT/BCIP. After WMISH, samples were optionally counter stained with SYTOX Green (Invitrogen; 450–490 nm) and imaged on a glass slide in PTW using a MZ FL III Stereo-Fluorescence Microscope (Leica, Wetzlar, Germany) equipped with a ProgResC3 sensor (Jenoptik, Jena, Germany).

## Results

### *caudal* localisation

**Fig 3:**
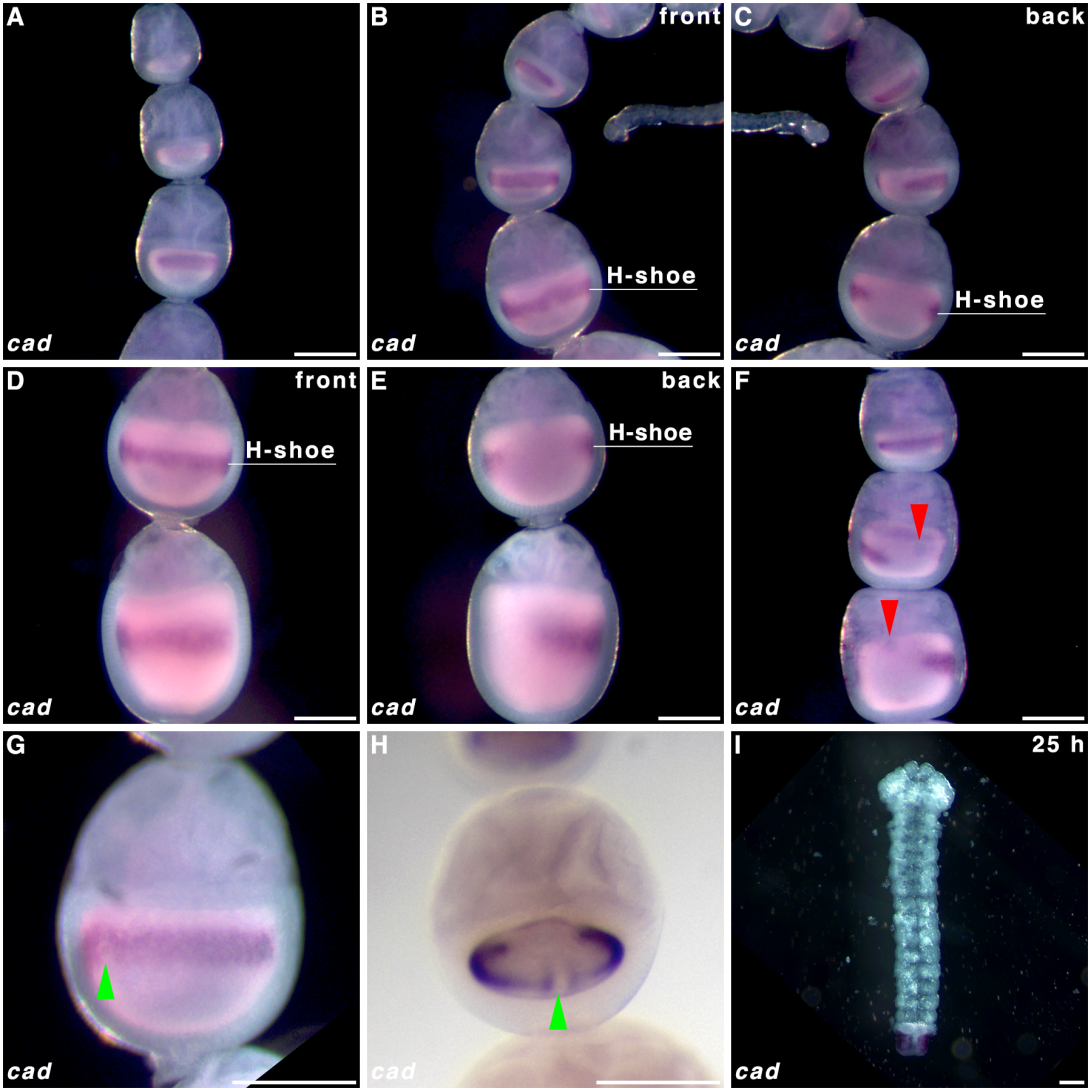
*caudal* transcript localisation in *Pararge aegeria* oocytes. Ovarioles were hybridised with a riboprobe targeting *cad* RNA **(A-H)**, and a 25 h embryo stained with the same riboprobe **(I)**. Panels **C** and **E** show the reverse of ovarioles in panels **B** and **D** respectively. Red arrows in **F** indicate the nucleus, which aligns with the gap in the ‘horseshoe’ **(H-shoe)** localisation pattern. Green arrows in G and H point to a clearance in the front (‘toe’) of the ‘horseshoe’ shaped pattern, which is exactly opposite to the gap at the back (‘heel’) of the ‘horseshoe’ shape. All times after egg-laying (AEL). Scale bars 200 μm.

**Fig 4:**
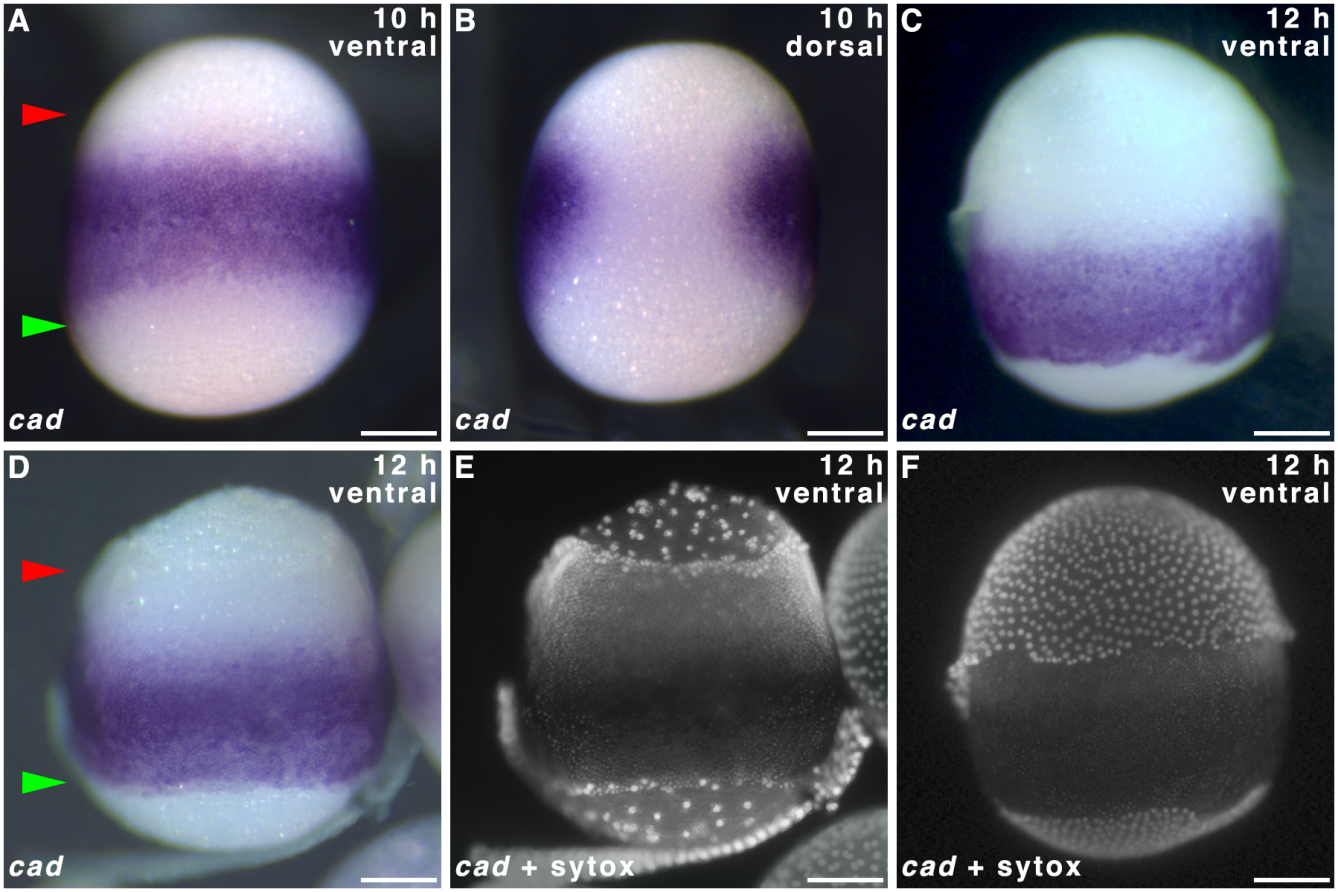
*caudal* expression in 10–12 h old *Pararge aegeria* embryos. 10 h **(A-B)** and 12 h **(C-F)** embryos hybridised with riboprobes staining *cad*. Panels **E** and **F** show Sytox green stain for embryos shown in **D** and **C** respectively. Note the serosa is removed in **E**. In **A** and **D** red and green arrows mark the anterior and posterior edges of the germ band respectively. All embryos are oriented with the anterior to the top. All embryos were observed from the ventral face except **B** (dorsal). All times after egg-laying (AEL). Scale bars 200 μm.

To determine whether *cad* forms a posterior gradient established maternally, we performed WMISH on oocytes in *P*. *aegeria* ovarioles and in the blastoderm of early embryos. Transcripts of *cad* form a ‘horseshoe-shaped’ band within the cortical region of the oocyte that will give rise to the embryonic region of the blastoderm (i.e. germ cortex; illustrated in green in Fig 2 and clearly demarcated from the extraembryonic region [1]). The *cad* localisation pattern emerges halfway through the primary growth phase in the ovarioles (Fig 3A; for growth phases see Fig 1). The edges of the *cad* ‘horseshoe’ domain do not appear sharply defined in the oocyte (Fig 3B–3E). As the oocyte matures, the *cad* band moves progressively from the anterior (Fig 3B and 3C) to a more central location in the oocyte (Fig 3D and 3E), which corresponds with the initial localisation observed in the germ band in early (10 h AEL) embryos (Fig 4A and 4B). The gap in the ‘horseshoe’ (‘heel’) (Fig 3C and 3E) marks the location of where the extraembryonic bridge joining anterior and posterior on the dorsal side of the embryo will form (see Figs 2B and 4B and [1]).

In a number of maturing oocytes the nucleus could be identified to the anterior of the ‘horseshoe heel’ (Fig 3F). Additionally a small clearance in the ventral side of the ‘horseshoe’ band (‘toe’) could be intermittently discerned (Fig 3G and 3H).

In 25-hr old embryos *en* transcripts were localised at the posterior of each segment, which is highly conserved among insects (Panels A and B S2 Fig; [30]). Transcripts of *cad* were detected in the posterior end of the abdominal region and telson (S2 Fig Panel C and Fig 3I).

### *Orthodenticle* localisation

**Fig 5:**
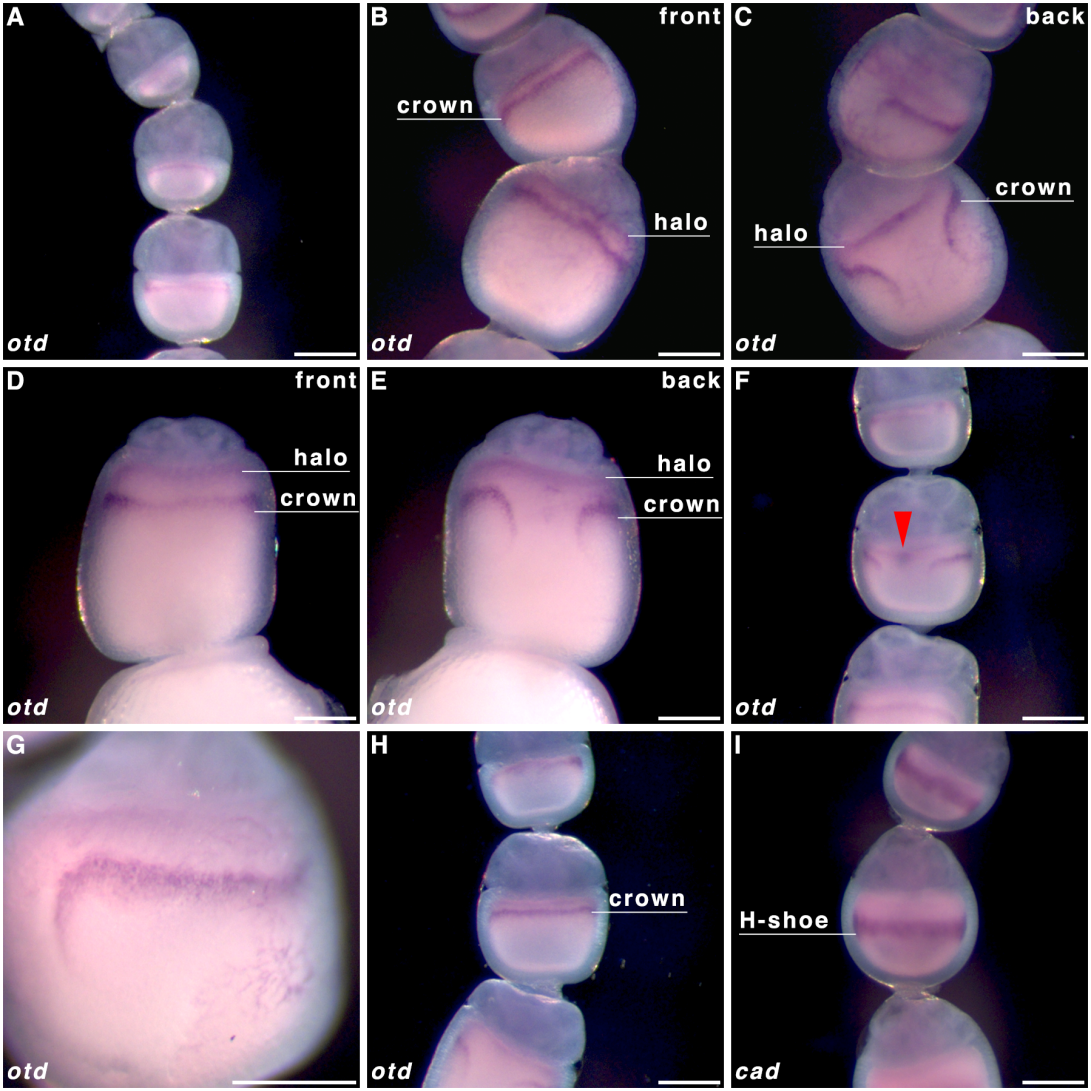
*orthodenticle* transcript localisation in *Pararge aegeria* oocytes. Ovarioles were hybridised with a riboprobe targeting *otd* RNA **(A-H)**. For comparison purposes *cad* localisation in an oocyte of a similar stage to **H** is also shown in panel **I**. Transcripts for *otd* localise to 2 distinct domains; an anterior domain **(halo)** lining the nurse cell-oocyte boundary and a more posterior domain **(crown)**; a narrow band that breaks after curving posteriorly on one face. Panels **C** and **E** depict the back view of the ovarioles in panels **B** and **D** respectively. All ovarioles are oriented in such a way that the AP axis in maturing oocytes is depicted top to bottom (i.e. anterior of oocyte is bordering the nurse cells). Scale bars 200 μm.

To determine whether *otd* could act as a maternally provided anterior determinant, we performed WMISH on oocytes in *P*. *aegeria* ovarioles. Transcripts of *otd* localise into a specific pattern soon after exiting the germarium (analogous to *cad*) (Fig 5A). The pattern is made up of two components, one static anterior diffuse ‘halo’ (Fig 5B) and one dynamic narrow semi-circular band, labelled the ‘crown’ which curves posteriorly at the breaking point (Fig 5C; see also 5D and 5E for comparison). The distance between the crown and the nurse cell-oocyte boundary (and ‘halo’) increases as oocytes mature (Fig 5A–5E). Observations also showed the anteriorly located nucleus appears on the same face as the gap in the *otd* ‘crown’ (as it does in the *cad* ‘horseshoe’) (Fig 5F). Like the *cad* ‘horseshoe’ pattern, the gap in the *otd* crown corresponds to the dorsal side. The anterior *otd* ‘halo’ boundary is initially fairly well defined (Fig 5B and 5C) but rapidly becomes more diffuse than the ‘crown’ (Fig 5D, 5E and 5G). Comparison of oocytes at very similar stages of oogenesis displaying the *cad* and *otd* localisation patterns indicated that the *otd* ‘crown’ is likely located to the anterior (Fig 5H) of the more central *cad* localisation domain (Fig 5I).

### *hunchback* in early embryos

**Fig 6:**
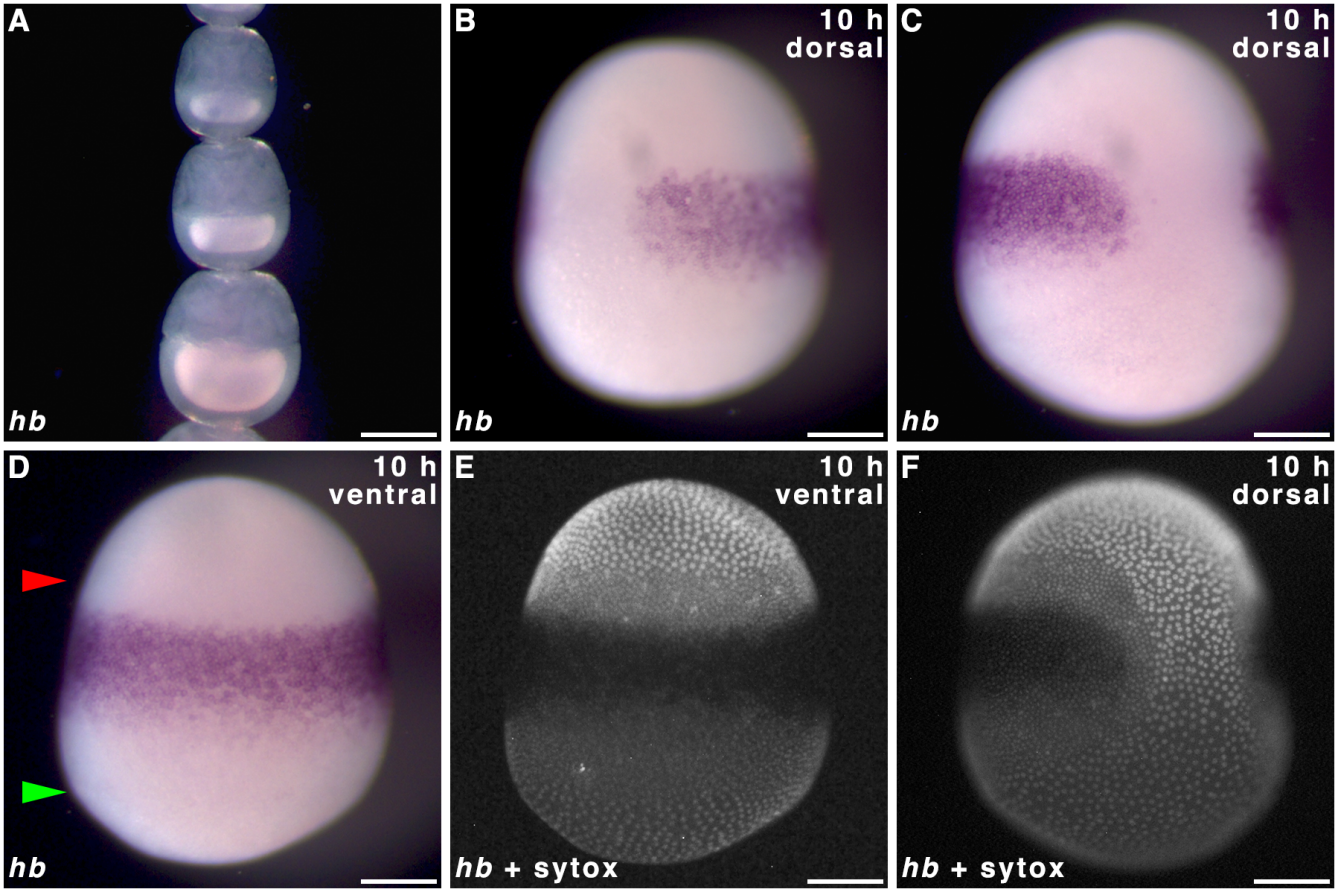
*hunchback* expression in *Pararge aegeria* ovarioles and embryos. Ovariole **(A)** and 10 h embryos **(B-F)** hybridised with riboprobes staining *hb*. Panels **E**, and **F** show Sytox green stain for embryos shown in **D** and **C** respectively. In **D** red and green arrows mark the anterior and posterior edges of the germ band respectively. All embryos are oriented with the anterior to the top. All embryos were observed from the ventral face except **B, C, F** (dorso-lateral). All times after egg-laying (AEL). Scale bars 200 μm.

To determine whether hunchback is localised maternally and whether expression in the blastoderm is in accordance with the classical gap gene role of *hb*, we performed WMISH on oocytes in *P*. *aegeria* ovarioles and the blastoderm of early embryos. No *hb* transcripts were detected in ovarioles (Fig 6A), which is in agreement with previous transcriptome findings [6]. Blastoderm cells of the germ band showed *hb* expression forming the familiar albeit narrower ‘horseshoe’ type band (Fig 6B–6F) with a gap located dorsally (Fig 6B and 6C) which makes way for the large extraembryonic fated cells that bridge anterior and posterior (Fig 6E and 6F; also see [1]). However the *hb* expression domain is restricted to the anterior-median of the germ band; cells near the anterior pole (between upper green arrow and upper red arrow in Fig 6D) and in most of the posterior-half of the germ band (between lower red arrow and lower green arrow in Fig 6D) did not express *hb* (Fig 6D–6F). The *hb* expression is also restricted from the very lateral domains of the germ band (adjacent to the extraembryonic bridge) (Fig 6F).

### Localisation of *nanos* paralogs

**Fig 7:**
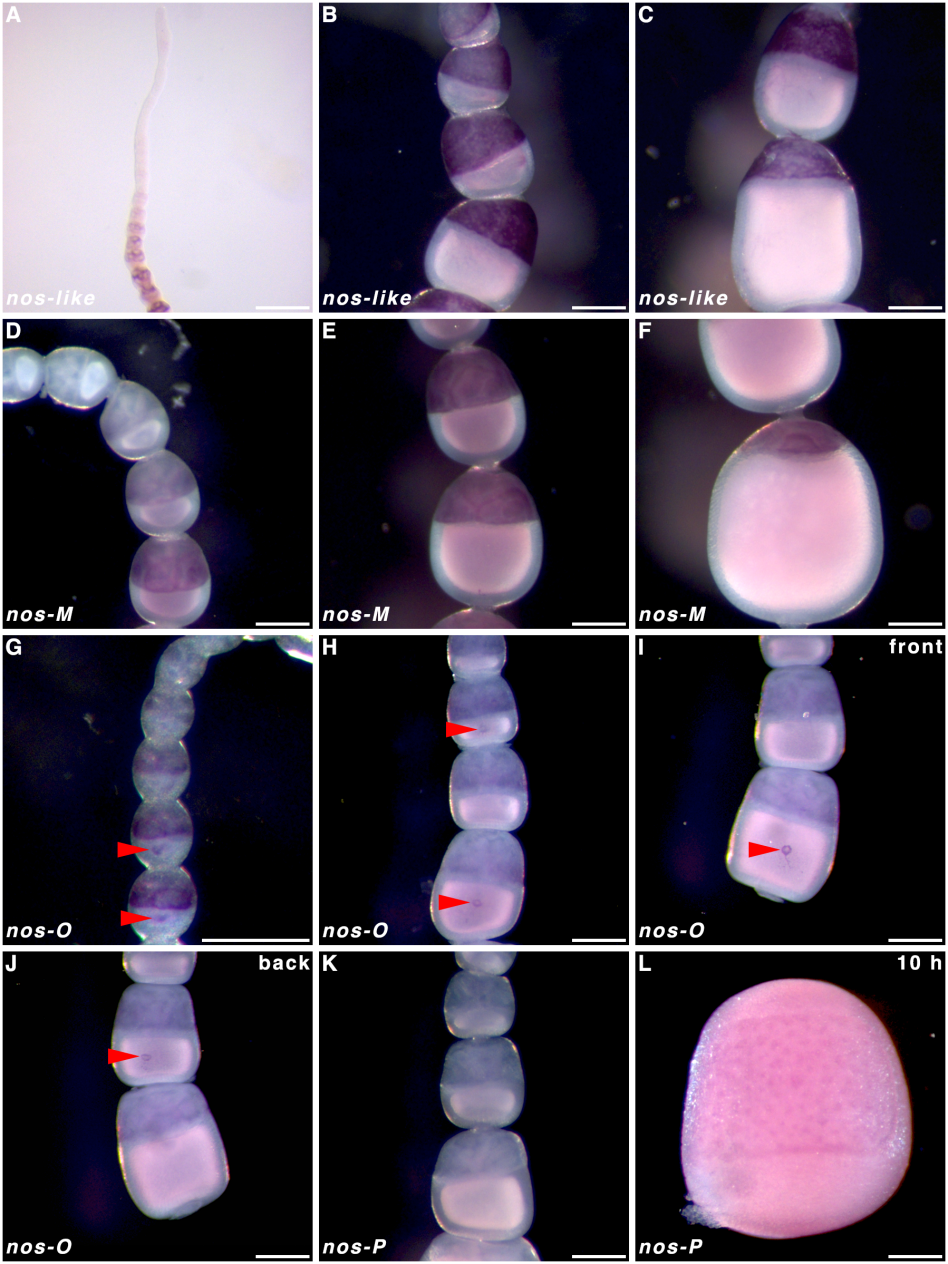
*nanos* expression and localisation in *Pararge aegeria* ovarioles and embryos. Ovarioles were hybridised with a riboprobe targeting *nos-like* **(A-C)**, *nos-M* **(D-F)**, *nos-O* **(G-J)** and *nos-P* **(K)** RNA. Embryo stained for *nos-P* transcripts **(L)**. Red arrows indicate *nos-O* transcripts localised in a small ring pattern on the ventral side of the oocytes **(G-J)**. The ovariole in **G** was overstained compared to **H** and **I**. Panel **J** shows the reverse of ovariole in **I**. All times after egg-laying (AEL). Scale bars 200 μm.

In order to infer the functionality of the various *nos* paralogs in AP patterning and possible germ plasm formation, we performed WMISH on oocytes in *P*. *aegeira* ovarioles and in early embryos. Three of the four *nos* paralogs were found to be expressed in the nurse cells of oocytes in ovarioles during oogenesis; *nos-like*, *nos-M* and *nos-O* (Fig 7A–7J). The only *nos* paralog not to be expressed maternally, but zygotically, was *nos-P* (Fig 7K and 7L;). However, *nos-P* transcripts did not appear to be localised in a recognisable pattern in the embryos (Fig 7L). Transcripts of *nos-like* were most strongly detected in the nurse cells of follicles leaving the germarium (Fig 7A) until nurse cell apoptosis (Fig 7B and 7C). However, transcripts were not detected in the oocytes. Similarly, *nos-M* expression was detected in the nurse cells albeit at lower levels and at a later point in the primary growth phases (Fig 7D–7F). Mild over-staining of ovarioles hybridised against *nos-O* transcripts showed both early expression and localisation of transcripts in the oocyte in ovarioles (Fig 7G). *Nanos-O* transcripts localised in a small ‘O’ or ring formation (under normal staining time) in the middle of the oocyte (Fig 7H–7J).

**Fig 8:**
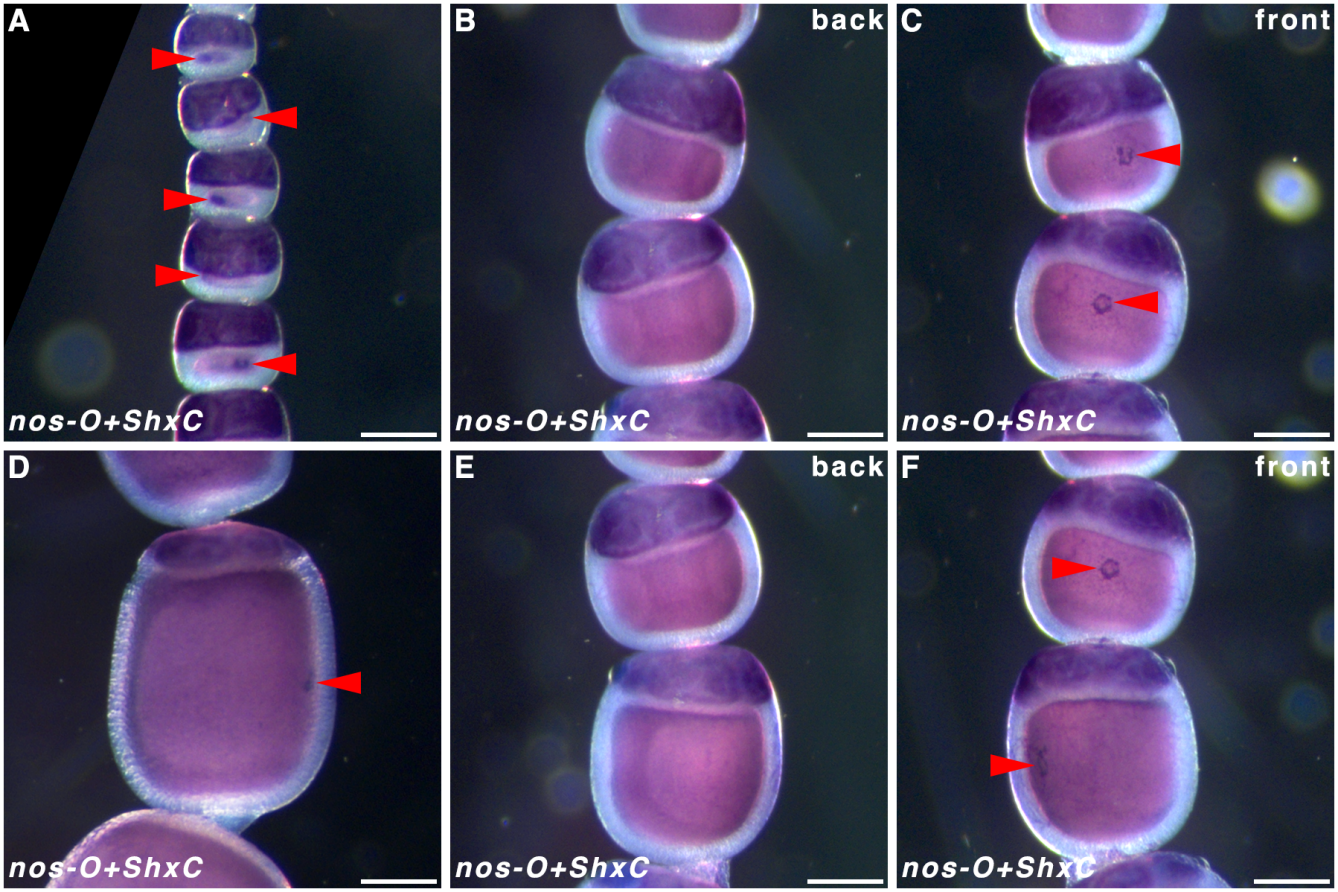
Relative localisation of *ShxC* and *nanos-O* in *Pararge aegeria* oocytes. Ovarioles hybridised with both riboprobes to reveal localisation of *nos-O* relative to *ShxC*. The typical *nos-O* granular ring is indicated with red arrows. **C** and **F** show reverse of ovarioles in **B** and **E** respectively.

Localisation of *ShxC* transcripts has been shown to provide a clear demarcation between the embryonic and extra-embryonic region [1]. To clarify where along the dorsoventral embryonic axis the maternal *nos-O* RNA localised we performed double WMISH using *ShxC* to visualise the extra-embryonic region, thus outlining where the germ band will form. Localisation of *ShxC* and *nos-O* were revealed to be consistently exclusive of one another (Fig 8). In particular, the *nos-O* ring forms (Fig 8A–8F) exactly opposite the dorsal extraembryonic bridge demarcated by the *ShxC* hourglass localisation pattern (Fig 8B and 8E)—effectively demonstrating that *nos-O* localisation is mid-ventral. This may also correspond to the ventral clearance in the *cad* horseshoe pattern previously described (Fig 3G and 3H).

### *Tdrd7* expression in *Pararge aegeria* follicles

**Fig 9:**
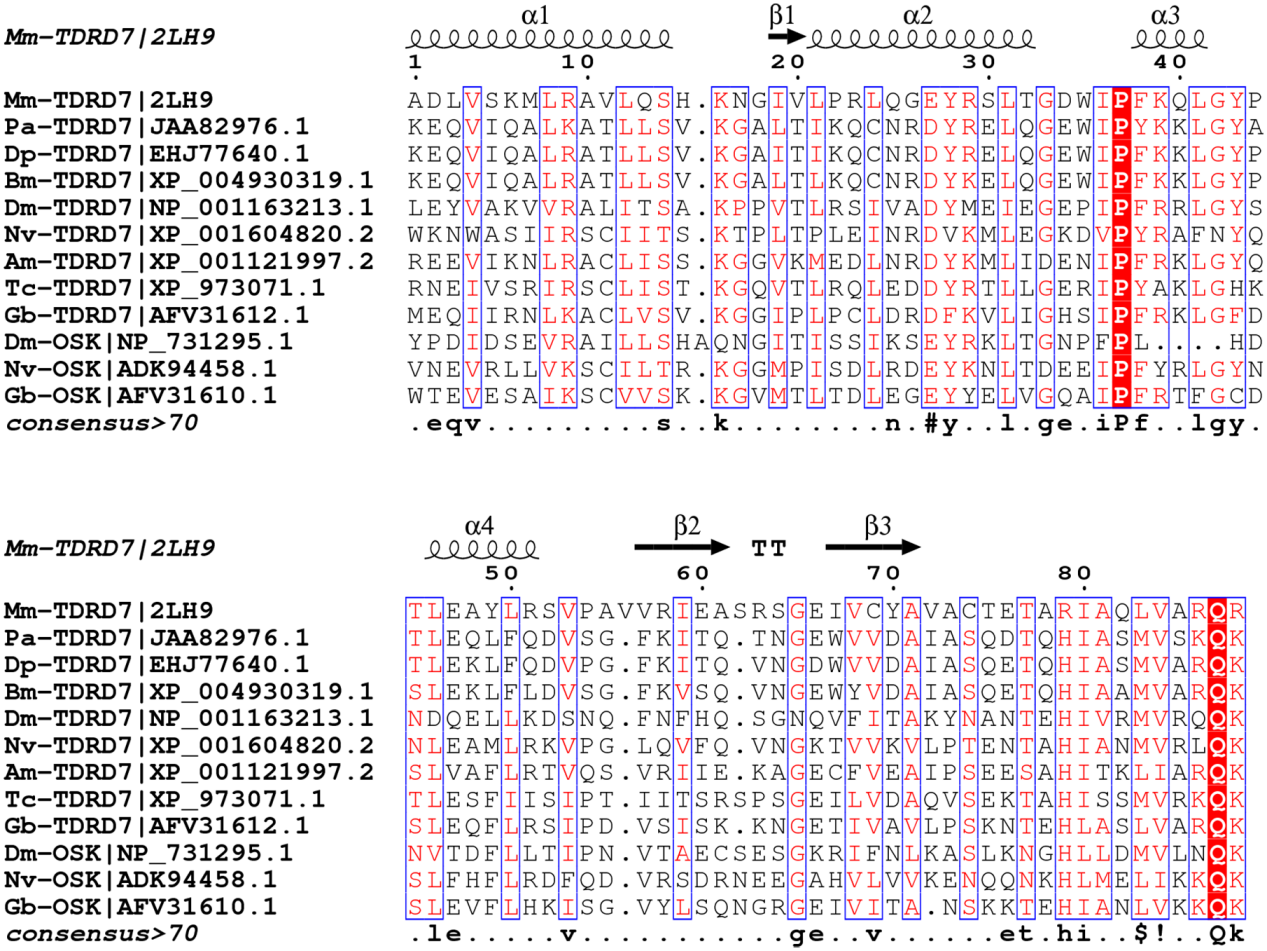
Alignment of the OST-HTH/LOTUS domain in TDRD7 and Oskar. Alignment of the conserved LOTUS domain shared by the TDRD7 and Oskar proteins from *Pararge aegeria* (*Pa*), *Danaus plexippus* (*Dp*), *Bombyx mori* (*Bm*), *Drosophila melanogaster* (*Dm*), *Nasonia vitripennis* (*Nv*), *Apis mellifera* (*Am*), *Tribolium castaneum* (*Tc*), *Mus musculus* (*Mm*) and *Gryllus bimaculatus* (*Gb*). Secondary structure annotation obtained from *M*. *musculus* structural data (PDB: 2LH9). Figure generated in ESPript 3.0 [57]. Columns with residues of similar physico-chemical properties (equivalent residue percentage) over 70% are in red with blue frames, strictly conserved positions highlighted red. The consensus sequence displays strictly conserved residues in uppercase while lowercase symbols indicate columns with a “MultAlin” similarity over 70% (IV / LM / FY / NDQEBZ). Exclamation mark refers to the amino acid I or V, “$”refers to the amino acid L or M, “%”refers to the amino acid F or Y, “#”refers to either the amino acid N, D, Q, E, B, or Z.

**Fig 10:**
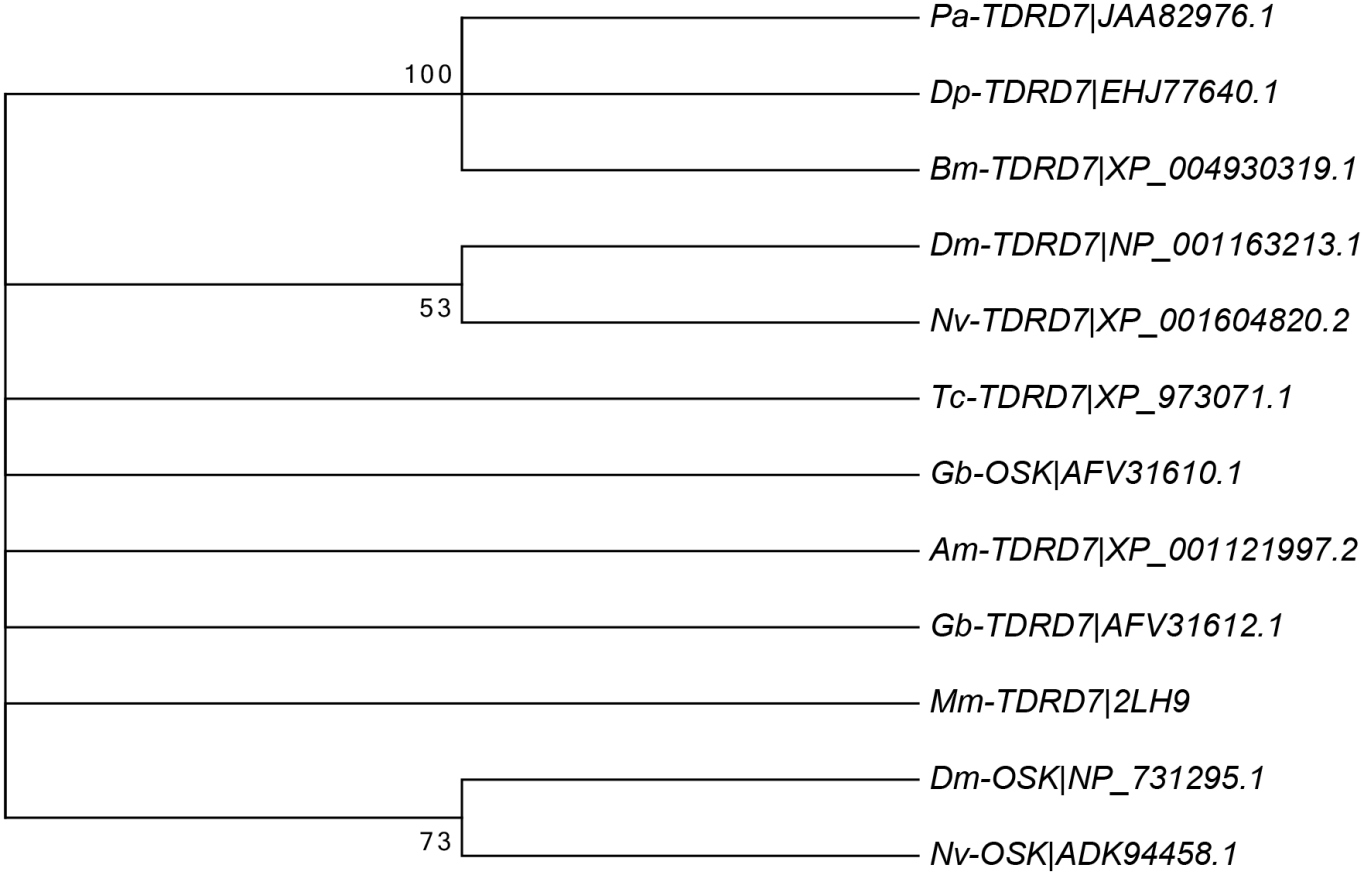
Phylogenetic analysis of the TDRD7 LOTUS domain. The evolutionary history for the OST-HTH/LOTUS domain shared by TDRD7 and Oskar proteins was inferred using the Neighbor-Joining method. The bootstrap consensus tree inferred from 2000 replicates is taken to represent the evolutionary history of the taxa analysed. Branches corresponding to partitions reproduced in less than 50% bootstrap replicates are collapsed. The percentage of replicate trees in which the associated taxa clustered together in the bootstrap test (2000 replicates) are shown next to the branches. The evolutionary distances were computed using the Jones, Taylor & Thornton matrix-based method and are in the units of the number of amino acid substitutions per site. The analysis involved 12 amino acid sequences. All positions with less than 95% site coverage were eliminated. That is, fewer than 5% alignment gaps, missing data, and ambiguous bases were allowed at any position. There were a total of 81 positions in the final dataset. Evolutionary analyses were conducted in MEGA6.

The alignment and phylogenetic analysis of the OST-HTH/LOTUS domain of TDRD7 and Oskar proteins in insects shows overall a high level of conservation for key amino acids, but not a significant distinction between TDRD7 and Oskar (Figs 9 and 10). It is likely that this domain is involved in RNA binding and thus for regulating mRNA translation and/or localisation in germ cell development [28].

**Fig 11:**
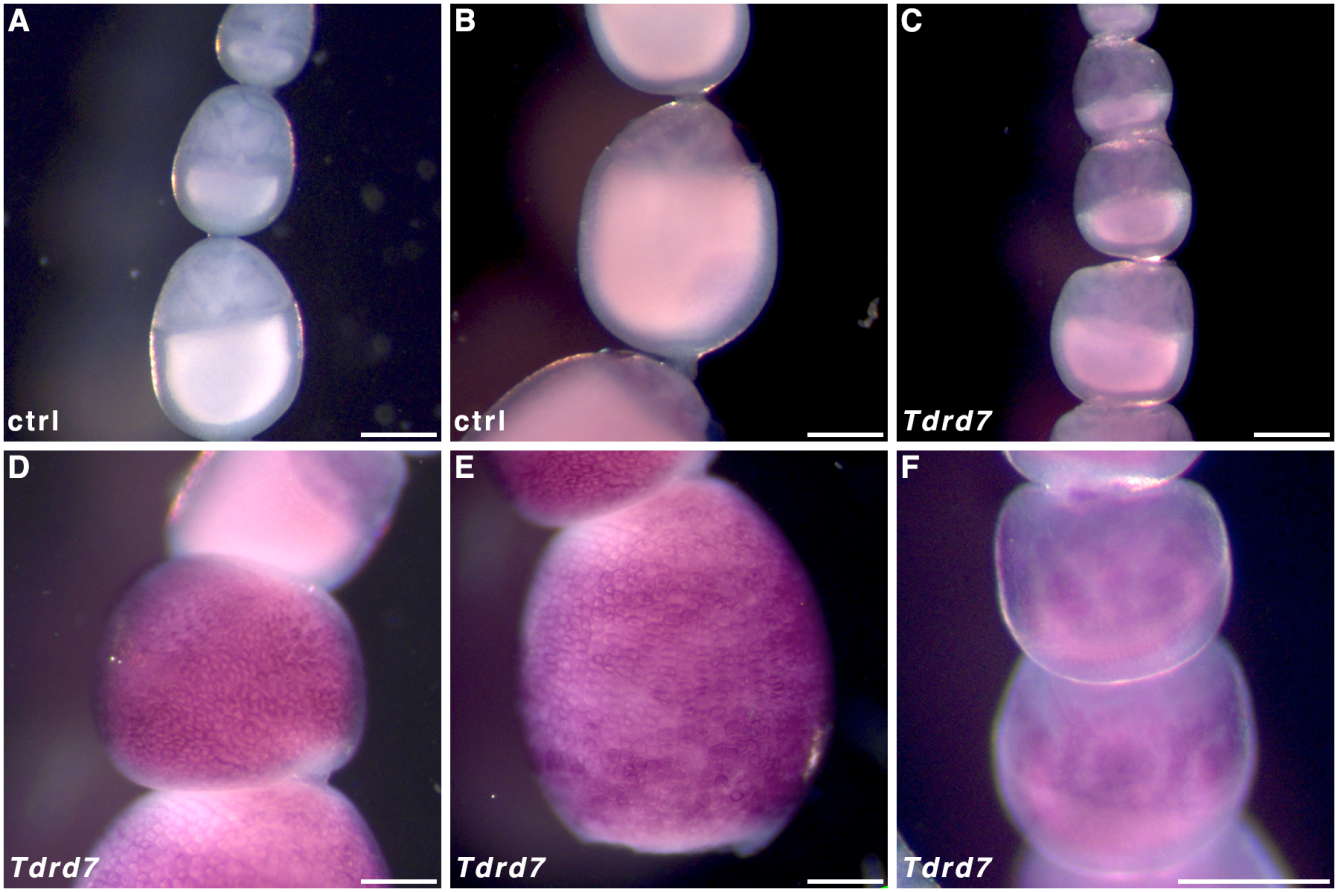
*Tdrd7* expression in *Pararge aegeria* ovarioles. *Pararge aegeria* ovarioles hybridised with control sense (**A** and **B**) and antisense riboprobes targeting Tdrd7 transcripts (**C-F**). Tdrd7 nurse cell expression is only visible after extensive staining **(F)**. Scale bars are equivalent to 200 μm.

In order to infer whether *Tdrd7* may be functionally similar to *osk* and thus localise where the PGCs will form (cf. *nos-O*), we performed WMISH on oocytes in ovarioles. *Tdrd7* transcripts were most prominently detected in follicle cells surrounding oocytes that have entered the chorion formation phase (Fig 1) of oogenesis (Fig 11). However, extended staining time did reveal some *Tdrd7* expression at earlier stages in the nurse cells (Fig 11F) relative to controls (Fig 11A and 11B), but not where transcripts of *nos-O* localise.

## Discussion

Metazoan embryos all develop PGCs, but there are significant differences in both timing and underlying developmental mechanisms [31,32]. These cells either result through inductive interactions between somatic cells during embryogenesis (i.e. epigenesis) or through a maternally produced germ plasm (i.e. preformationism) [31,32]. It appears that epigenesis is the ancestral state and that the maternally regulated germ plasm formation has arisen multiple times over evolutionary time through convergent evolution [33]. Basal insects rely on epigenesis and a number of derived insects have been shown to possess a germ plasm [5]. The deployment of *osk* in germ plasm formation is considered an evolutionary novelty in insects [5,15].

Butterflies are in many aspects of their development highly derived, and based on studies of Ditrysian moths, they have been argued to not have a germ plasm [5]. To date, no Lepidopteran *osk* orthologs have been detected, including in *P*. *aegeria* [6]. Molecular markers for the germ line (*vasa*) can, however, already be detected in the *B*. *mori* oocyte [34]. Localisation only occurs later in the embryonic blastoderm and presents an argument against the existence of a germ plasm [34,35]. Although *nos* is not a key determinant of the germ plasm in holometabolous insects, three *nos* paralogs were maternally expressed: *nos-O*, *-like*, and–*M* similarly to *B*. *mori* [10]. In *B*. *mori*, localisation of *nos-O* transcripts ventrally in the pre-blastoderm is variable [10]. In *P*. *aegeria* however, *nos-O* transcripts accumulate in a distinct ring in the ventral cortex. This site of *nos-O* transcript accumulation matches even more precisely to the ventrally formed germ cells that characterise the Ditrysia [34,36] and thus may presumably play a key role in PGC formation, and possibly its specification.

The other two maternally expressed *nos* paralogs, *nos-like* (also known as *nos-N*), and *nos-M* appear to be heavily transcribed in the nurse cells, but no obvious localisation of these transcripts in the maturing *P*. *aegeria* oocytes could be detected. Judging by the intensity of the staining observed, *nos-like* was most strongly transcribed followed by *nos-O*, and *nos-M*, which is in agreement with previous RT-qPCR data [6]. The apparent lack of a visible accumulation in the maturing oocytes of *nos-like* and *-M* is perhaps surprising for two reasons; 1) maternal transcripts were detected for these *nos* paralogs in a freshly laid egg [6], and 2) Nakao et al. [10] were able to detect both transcripts in the oocytes prior to embryogenesis. Furthermore, *nos-like* has been shown to be enriched in the germ cortex of *B*. *mori* eggs [10]. Perhaps such localisation is only initiated in the very final phase of oogenesis when chorion and vitelline membrane formation make riboprobe penetration difficult [6]. Further experiments are required to gain any decisive insight into the function of *nos-like* and *nos-M* during *Pararge* oogenesis and their possible contribution to the regulation of the early stages of embryogenesis.

The gene *osk* appears to be rapidly evolving and a significant amount of sequence variability has been detected in holometabolous insects, with Drosophilid *osk* being characterised by a so-called Long Oskar domain [29]. A number of functional domains characterise *osk* in holometabolous insects in general including a particular RNA binding domain (OST-HTH/LOTUS) [27,28], which has also been found in the Tudor-family gene *Tdrd7* [5,15,29] (see also Figs 9 and 10). Given that Ditrysia appear to have lost *osk* it could be hypothesised that *Tdrd7* may have evolved similar functionality to *osk* in Ditrysia. This gene was found to be expressed during *P*. *aegeria* oogenesis [6]. However, no specific oocyte localisation was observed. Further work is required to determine if *Tdrd7* could possibly play a functional role in either PGC specification or formation. Despite the *nos-O* localisation it is still unresolved whether Ditrysia have completely dispensed with a typical germ plasm when they lost *osk*.

Phylogenetic analysis of *nos* sequences showed *nos-P* to be highly divergent from the other paralogs [6], suggesting it may have a different functional role. Although minor ovarian *nos-P* expression has been detected in *B*. *mori* [10], we were unable to detect *nos-P* by means of *in situ* hybridisation or transcriptomics [6]. Additionally, Speckled Wood embryos did not show significant zygotic expression for *nos-P* at 10 h AEL. It may be that the onset of *nos-P* expression in butterflies is delayed compared to other early zygotic transcription and *Bombyx* [10]. Nevertheless it seems unlikely that *nos-P* represents a major maternal contribution to the regulation of silkmoth or butterfly early embryogenesis.

The localisation patterns for *cad* and *otd* in the maturing oocytes show a characteristic semi-circular type pattern (‘horseshoe’ and ‘crown’ respectively) around the central periphery. Additionally the nucleus (indicated in Figs 3F and 5F, but also visible in unstained oocytes in ovarioles) was always found to the anterior of the gap in the *otd* crown and or *cad* ‘horseshoe’ patterns, suggesting a dorsal-anterior localisation. Both the ‘crown’ component of *otd* and the ‘horseshoe’ of *cad* distanced themselves from the nurse cell-oocyte border (anterior). This may indicate that the transcripts moved posteriorly or the anterior half of the oocyte grew in size relative to the rest of the oocyte. The complex localisation patterns for these transcripts are very divergent from the patterns observed in the holometabolous model species *Drosophila*, *Tribolium* and *Nasonia* [4,17,20,30,37,38]. Embryonic RNAi knockdown in *Bombyx* orthologs for *otd* and *cad* has indicated that expression of these genes is essential for AP patterning [11]. Although butterflies currently lack RNAi, the localisation patterns reported here clearly show how precisely and early this information must be set-up by the mother to direct patterning of the embryonic blastoderm.

Work in *Tribolium* has questioned whether *otd* has an ancestral role in anterior patterning [20–22], and highlighted the relevance of other patterning genes such as *mex-3* [23]. However, the distinct anterior localisation of maternal *otd* RNA in *Pararge*, as well as *Bombyx* experiments [11], strongly suggest a role for maternal *otd* in regulating anterior patterning in Ditrysia. Although preliminary data show the KH domain factor *mex-3* is expressed during oogenesis and included as transcripts in *P*. *aegeria* oocytes [6], preliminary *in-situ* data (not shown) is inconclusive as to whether it is functionally localised; i.e. in a role with respect to Cad protein localisation. It is therefore still uncertain whether *mex-3* has a role in AP patterning in butterflies.

The results reported here confirm that butterflies, just like moths, express *hb* zygotically [11]. Zygotic expression of *cad* and *hb* showed overlapping expression domains in the germ cortex indicating they do not repress each other in agreement with functional studies in *Bombyx* [9,11]. The central localisation of maternal *cad* transcripts suggests an intriguing, but as yet unknown, maternal role. It is unclear whether the later posterior shift in embryos (12 h) is a result of zygotic expression or migration of cells containing maternal transcripts. Later in development, *cad* expression is observed in the telson, which suggests similar functionality in body patterning as seen in other insects [38].

### Cortical localisation and specification

As observed previously for *ShxC* [1], transcripts of *P*. *aegeria cad*, *otd*, and *nos-O* were very specifically localised in the oocyte cortex during oogenesis, prefiguring their roles in the cellularised blastoderm stage. These localisation patterns clearly demarcate an existing cortical boundary between the future regions of the blastoderm that will form germ (embryonic) and extraembryonic anlagen. This boundary therefore demarcates 2 cortical regions: the germ cortex and extraembryonic cortex.

**Fig 12:**
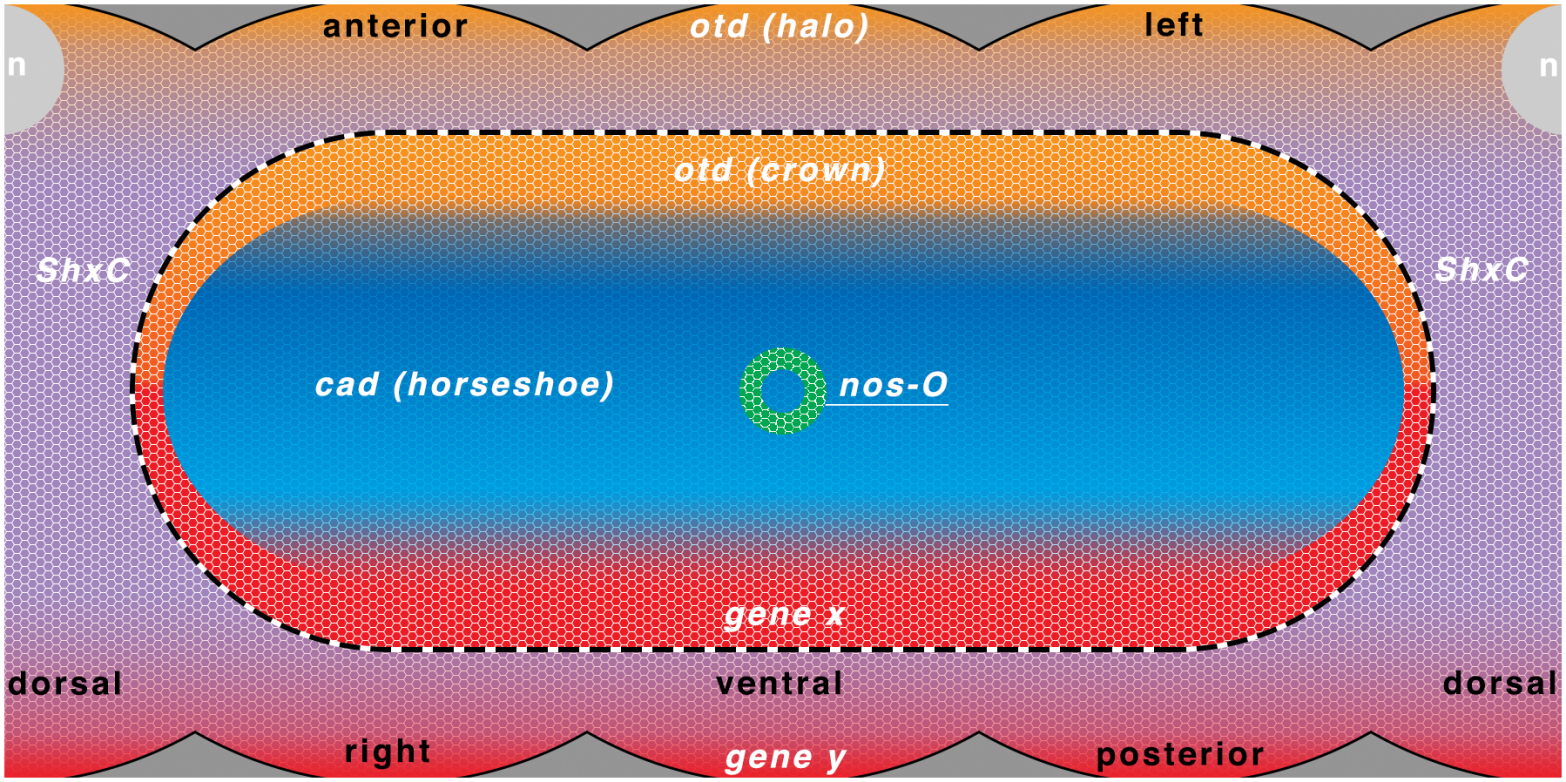
Diagram of cortical localisation domains for the genes investigated in *Pararge aegeria*. Simplified diagram of cortical localisation domains for *cad* **(blue)**, *nos-O* **(green)**, *otd* **(orange)** and *ShxC* **(purple)**. Domains in red are hypothetical examples of where other maternal effect genes may localise to participate in maternal regulation. Notably a posterior gradient to coordinate *caudal* localisation **(gene x)** and a factor to specify the posterior of the germ cortex/band **(gene y)** may be required. The nucleus when in antero-dorsal position is also illustrated **(n)**. Anterior is top and the ventral face is centre, while the dorsal face is split to the left and right (as indicated in black text). Germ cortex and extraembryonic cortex boundary is indicated by a dotted line.

The precise *ShxC* and *nos-O* mRNA localisation to the cortex also did not appear homogenous and instead showed a mesh-like and granular texture respectively indicative of some form of structured anchoring [1]. On the other hand, *cad* and *otd* localisation showed both gradient and anchoring characteristics. These regions and localisation domains are summarised in Fig 12.

The insect cortex (or periplasm) is an area at the periphery of the oocyte free of yolk and corresponds to where the fertilised zygotic nuclei migrate to during cleavage, as has been shown in *Drosophila* [39]. The cortex contains a cytoskeletal framework that has an important role in anchoring maternal effect gene transcripts in a number of studied insect species [40,41]. Most notably, actin filaments play a prominent role in mediating the localisation of pole plasm components including *osk* and *nos* RNA in *Drosophila* [42,43]. The specific mechanisms that mediate the direct anchoring of the RNA are still being investigated. It may even be that some transcripts themselves act as pseudo structural elements anchoring other RNAs to the cortical framework [44,45]. In Lepidoptera such an actin-rich cytoskeleton framework in the cortex has been identified in several species of Giant moths [46]. Furthermore, it has been shown that certain maternal mRNAs are capable of being strongly associated with this cortical structure [47]. Such a cortical cytoskeleton is therefore also likely to be involved in the precise maternal transcript localisation patterns observed in *P*. *aegeria*.

Such extensive anchoring is compatible with the coexistence of so-called “permissive” and “instructive” regulation [30,48]. Normally, permissive regulators are involved in regulating the distribution or activity of elements (RNA or protein) conveying instructive gradients, which upon translation have a regulatory role at the transcriptional level (i.e. switching genes on or off). For example, the Bicoid gradient holds both permissive and instructive roles in patterning the AP axis [30,49–52]. Interestingly, the dual pattern presented by *Pa-otd* features both a diffuse anterior pole (halo) and a precise germ cortex localisation (crown), this may suggest a spatial segregation of early acting permissive and late acting instructive functions for the same gene. In this scenario the halo may result from anterior diffusion and translation leading to an anterior permissive gradient while the *Pa-otd* RNA crown bears the instructive role that will be applied upon formation of the blastoderm.

Permissive protein gradients may also be directing or assisting in the anchoring of these RNAs to form these complex patterns. To form a pattern such as the *Pa-cad* ‘horseshoe’ would require a minimum of 3 permissive gradients one from the posterior (“gene x” in Fig 12), one from the anterior (possibly the *otd* halo gradient Fig 12) and optionally one dorsal unless anchoring is competitive, i.e. the area is occupied by *ShxC* in the extraembryonic cortex.

The extensive use of RNA anchoring may be directed and assisted by a secondary mechanism originating from the follicular epithelium. During oogenesis, the follicle cells participate in setting up the polarity of the follicle and generate or transfer LLTPs from the hemolymph during vitellogenesis. Intriguingly the fat bodies also supply actin to the oocyte in a similar way in Ditrysian Giant moths [46]. The follicle cells may therefore respond to the (as yet unknown) polarisation signals of the egg chamber by contributing an extra factor to the endocytic packages that disassemble in the oocyte cortex. Possibly allowing association with the local cortical cytoskeleton to specifically recruit transcripts or ribonucleic protein complexes produced by the nurse cells.

This complex cortical localisation of maternal RNA in Lepidoptera may explain why damaging the egg cortex of fertilised moth eggs (before cellularisation) caused segmental defects in subsequent embryos [53]. This fixed pre-cellular patterning is also compatible with the observation that Ditrysians blastoderm cellularisation lacks a typical syncytial stage with cleavage forming through asynchronous budding rather than invagination (furrows) [14,53]. A process that may employ mechanisms shared with pole cell formation in *Drosophila* [54,55].

Although it appears unlikely that the transcripts forming the *Pa-otd* crown, *Pa-cad* ‘horseshoe’ or *Pa-ShxC* hourglass are performing any permissive roles during oogenesis, these complex 3 dimensional patterns are precisely incorporated upon cellularisation and likely interpreted for instructive function by the newly formed blastoderm.

Our work has provided a much-needed first step in determining the spatio-temporal expression and localisation patterns for *cad*, *otd*, *hunchback* (*hb*), *Tdrd7*, as well as the *nos* paralogs *nos-like*, *-M*, *-O*, *and—P* in *P*. *aegeria* ovarioles and early embryos. Further work using similar and more disruptive techniques such as RNAi is necessary to understand how oogenesis and maternal control of early embryogenesis has evolved in butterflies and how it accomplishes the intricate cortical RNA localisation observed.

## Supporting Information

S1 Fig Overview of amplification sites for target sequences. A schematic overview of the transcript sequences for the genes investigated. Primer binding sites are illustrated at the ends of amplification regions for riboprobe generation. *Only a partial sequence was available for engrailed. See S1 Table for primer sequences.
(DOCX)

S2 Fig *engrailed* and *caudal* expression in *Pararge aegeria* embryos (phylotypic stage). Riboprobes targeting en (A and B) and cad (C) transcripts hybridised to P. aegeria embryos around the phylotypic stage. In situ hybridisations were performed on devitellinised embryos still wrapped around the yolk (A) and on embryos with the yolk dissected away (B and C). Embryo ages are in hours after egg-laying. Scale bars 200 μm.
(DOCX)

S1 Table Primer Sequences. Primer combinations for primary (RPT), antisense (AS-RP) or sense (S-RP) riboprobe template generation; annealing temperatures in degrees Celsius (Ta) and amplicon size in base pairs (bp) for each pairing.
(DOCX)
