# Supplementary material for: Divergent RNA Localisation Patterns of Maternal Genes Regulating Embryonic Patterning in the Butterfly Pararge aegeria
Source: PLoS One. 2015 Dec 3;10(12):e0144471. doi: 10.1371/journal.pone.0144471 (PMC4669120; doi:10.1371/journal.pone.0144471)

### Supporting Information - Figure S1. Overview of amplification sites for target sequences

A schematic overview of the transcript sequences for the genes investigated. Primer binding sites are illustrated at the ends of amplification regions for riboprobe generation. *Only a partial sequence was available for *engrailed.* See Table S1 for primer sequences.


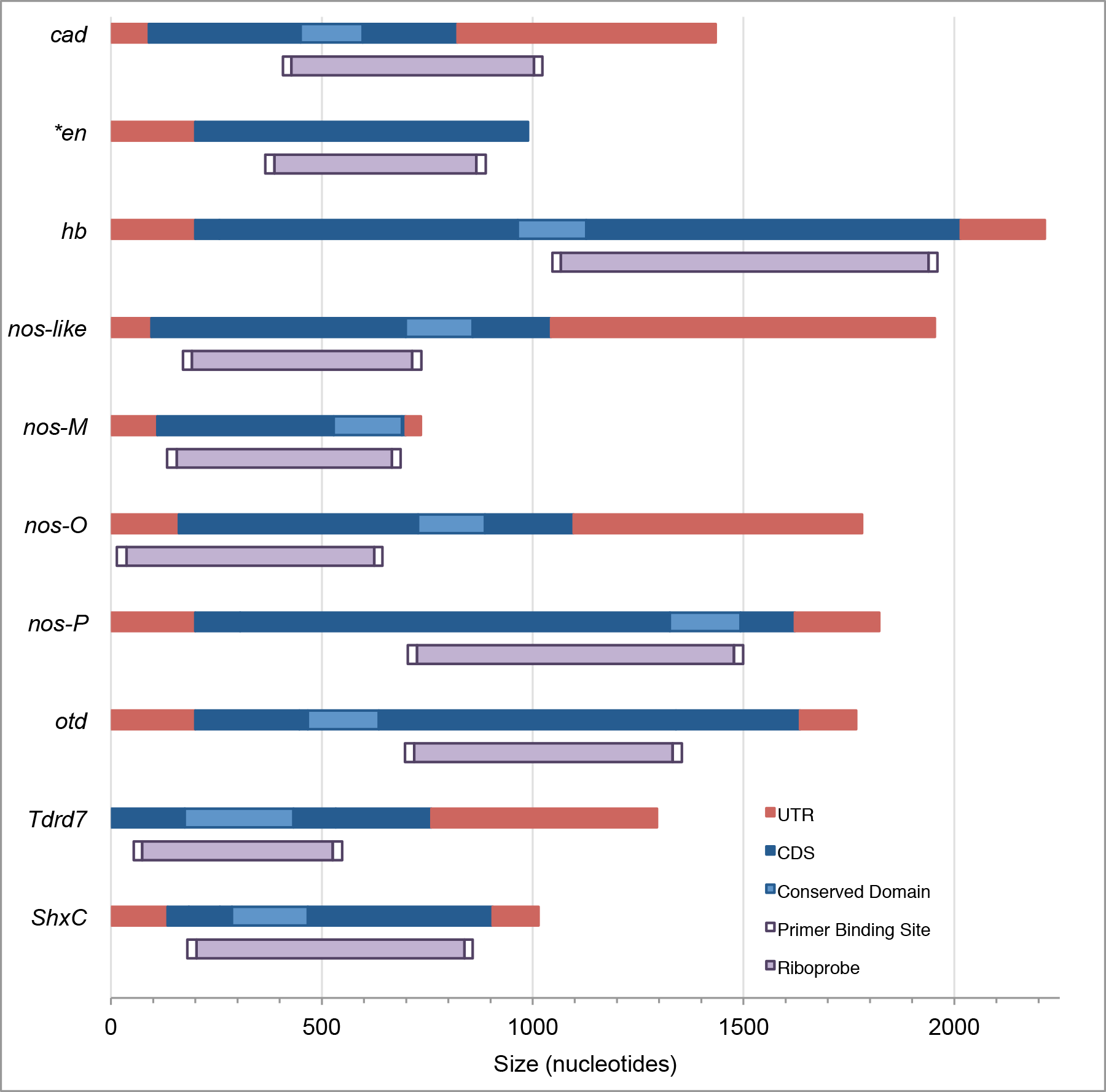

Supplement: S1 Fig — A schematic overview of the transcript sequences for the genes investigated. Primer binding sites are illustrated at the ends of amplification regions for riboprobe generation. *Only a partial sequence was available for engrailed. See S1 Table for primer sequences. (DOCX) [file pone.0144471.s001.docx]
