# Supplementary material for: Divergent RNA Localisation Patterns of Maternal Genes Regulating Embryonic Patterning in the Butterfly Pararge aegeria
Source: PLoS One. 2015 Dec 3;10(12):e0144471. doi: 10.1371/journal.pone.0144471 (PMC4669120; doi:10.1371/journal.pone.0144471)

### Supporting Information - Figure S2. *engrailed* (*en*) and *caudal* (*cad*) expression in *Pararge aegeria* embryos (phylotypic stage)

Riboprobes targeting *en* (A and B) and *cad* (C) transcripts hybridised to *P. aegeria* embryos around the phylotypic stage. *In situ* hybridisations were performed on devitellinised embryos still wrapped around the yolk (A) and on embryos with the yolk dissected away (B and C). Embryo ages are in hours after egg-laying. Scale bars 200 µm.


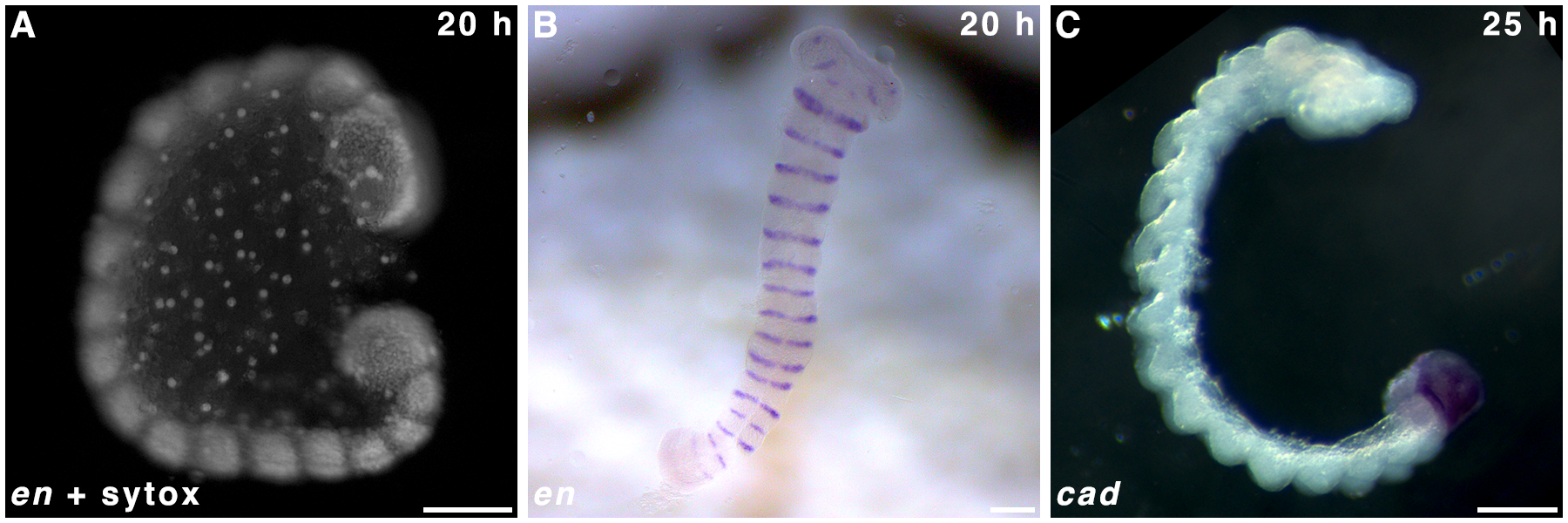

Supplement: S2 Fig — Riboprobes targeting en (A and B) and cad (C) transcripts hybridised to P. aegeria embryos around the phylotypic stage. In situ hybridisations were performed on devitellinised embryos still wrapped around the yolk (A) and on embryos with the yolk dissected away (B and C). Embryo ages are in hours after egg-laying. Scale bars 200 μm. (DOCX) [file pone.0144471.s002.docx]
