# Supplementary material for: Divergent RNA Localisation Patterns of Maternal Genes Regulating Embryonic Patterning in the Butterfly Pararge aegeria
Source: PLoS One. 2015 Dec 3;10(12):e0144471. doi: 10.1371/journal.pone.0144471 (PMC4669120; doi:10.1371/journal.pone.0144471)

### Supporting Information - Table S1. Primer Sequences

Primer combinations for primary (RPT), antisense (AS-RP) or sense (S-RP) riboprobe template generation; annealing temperatures in degrees Celsius (Ta) and amplicon size in base pairs (bp) for each pairing.


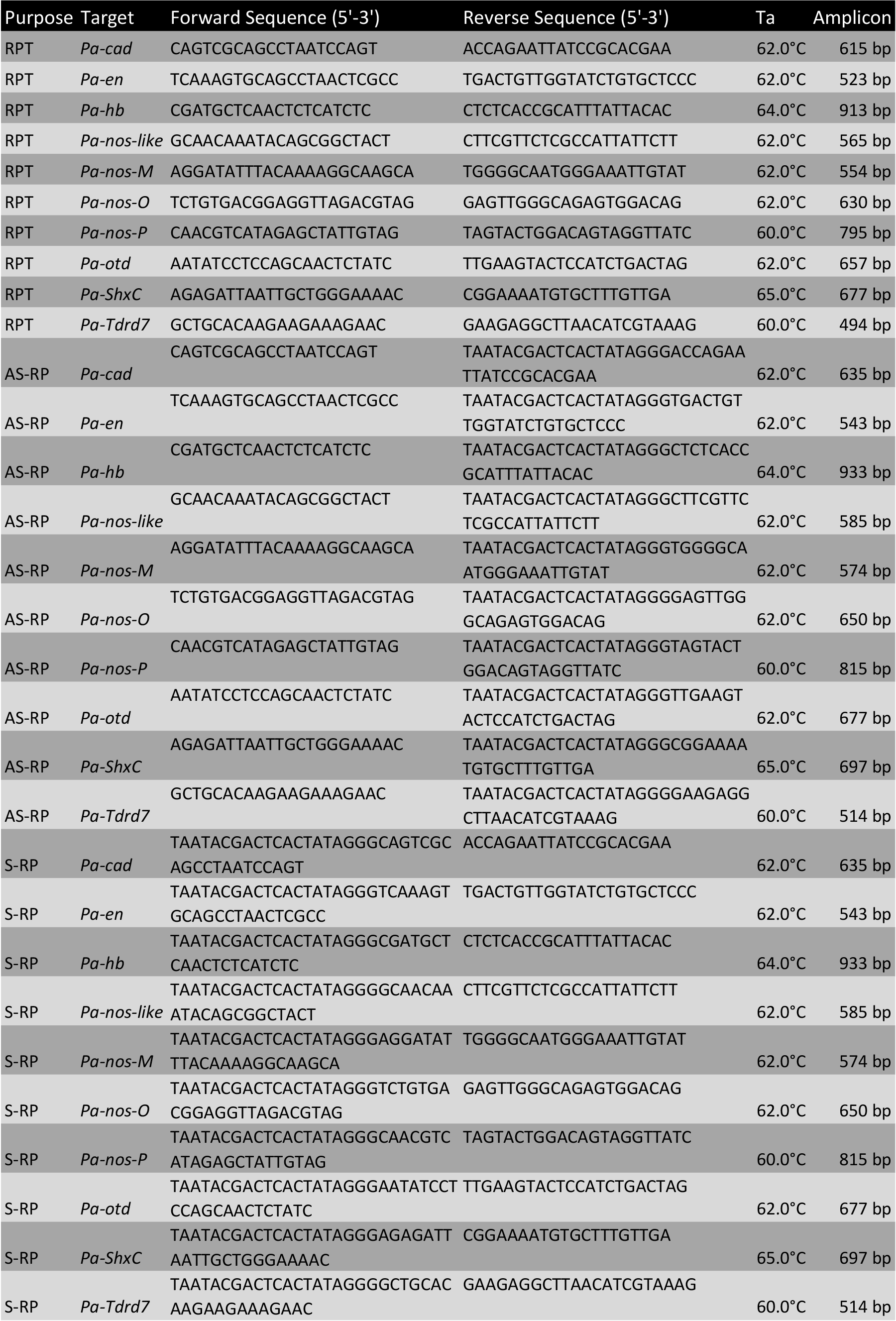

Supplement: S1 Table — Primer combinations for primary (RPT), antisense (AS-RP) or sense (S-RP) riboprobe template generation; annealing temperatures in degrees Celsius (Ta) and amplicon size in base pairs (bp) for each pairing. (DOCX) [file pone.0144471.s003.docx]
